# Villoglandular papillary adenocarcinoma: case report

**DOI:** 10.11604/pamj.2016.25.232.10305

**Published:** 2016-12-08

**Authors:** Ghizlane Salek, Issam Lalya, Driss Moussaoui Rahali, Mohamed Dehayni

**Affiliations:** 1 Department of Gynecology Obstetrics, Military Training Hospital Med V, Rabat, Morocco; 2 Department of Radiotherapy, HMIMV, Rabat, Morocco

**Keywords:** Villoglandular, subtype of adenocarcinoma, young women

## Abstract

Villoglandular papillary adenocarcinoma (VPA) is a very rare subtype of adenocarcinoma of the uterine cervix, but a well-recognized variant of cervical adenocarcinoma with a favorable prognosis and generally occurring in women of child-bearing age. Herein, we report a case of VPA diagnosed and managed successfully with conservative measure. This management is particularly desirable in young women to preserve reproductive capability.

## Introduction

The incidence of cervical adenocarcinoma is on the rise over the last decades. Villoglandular papillary adenocarcinoma (VPA) is a very rare subtype of adenocarcinoma of the uterine cervix. The true incidence of this form of adenocarcinoma is unknown. The classical histologic appearance of this entity is a surface papillary component of variable thickness with papillae that are usually tall and thin, but occasionally short and broad, with a fibrous stromal core. The tumor cells should have no more than mild-to-moderate nuclear atypicality and scattered mitotic figures. It affects a younger age group and has an excellent prognosis as compared to other endocervical adenocarcinomas [1, 2].

## Patient and observation

**Figure 1:**
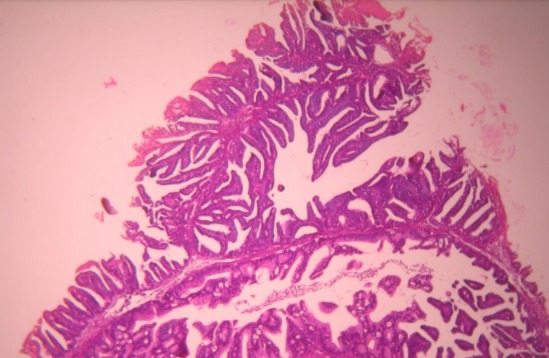
Hematein eosin x 5 villoglandular adenocarcinoma composed of papillary and glandular structures lined with cylindrical cells sometimes mucosecreting

**Figure 2:**
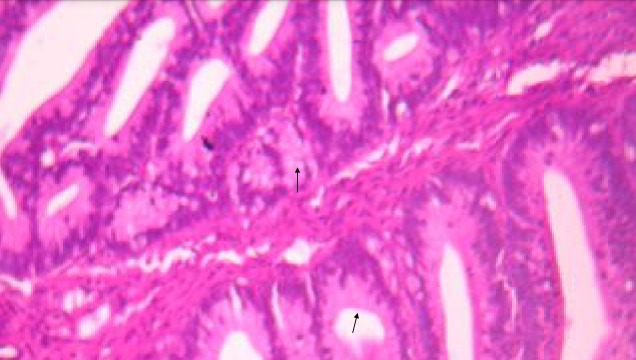
HEx200 ADK villoglandular: glands lined with mucosecreting cylindrical cells (arrows) without invasive element

**Figure 3:**
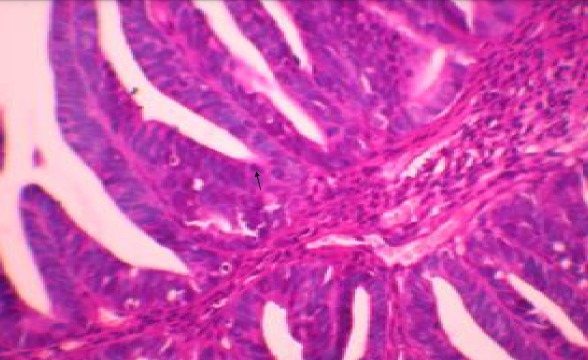
HEX400 details of papillary structures lined with columnar cells with moderate atypia with few mitotic figures (arrow)

A 29 year old para 1 presenting with a history of postcoital bleeding for three months. She was not using contraceptives and was trying for a pregnancy. On per speculum examination, a suspicious growth on the posterior lip of the cervix was noted for which a punch biopsy was done. The histo-pathological examination showed moderately differentiated papillary adenocarcinoma with a villoglandular pattern (Figure 1, Figure 2, Figure 3). There was no evidence of lymphovascular space invasion. An MRI showed a 3.2 × 2.5 × 3.6 cm tumour arising from the anterior lip of the cervix, with distal parametrial involvement but no significant pelvic or retroperitoneal lymph node enlargement. The multi-disciplinary team meeting confirmed a stage IIB distal cancer of the cervix and decided to treat with chemo-radiotherapy. She received 45 Gy in 25 fractions of external beam radiotherapy to the pelvis with concomitant weekly cisplatin 40 mg/m2. The radiotherapy dose to the primary cervical tumour was 26 Gy to point A. The primary cervical tumour had responded well to the initial chemo radiotherapy. After 4 weeks the cervical lesion has completely responded.

## Discussion

Adenocarcinomas are the second most common type of cervical cancers in women comprising 10-20% of the cases. Villoglandular papillary adenocarcinoma (VGA) of cervix is a rare but well recognized histologic subtype of cervical adenocarcinoma. It tends to appear in younger women [1] and its indolent behavior permits fertility-preserving treatments. VGA was first reported by Young and Scully in 1989, who described three main histological features: exophytic proliferation, papillary architecture and mild to moderate cellular atypia [1]. The etiology of villoglandular papillary adenocarcinoma has not been well established [3]. An association between the use of oral contraceptives and villoglandular papillary adenocarcinoma was suggested by Jones et al. Although there are reports of an association between the use of oral contraceptives and cervical adenocarcinoma, a specific association is not found between the reproductive function of the patient and cervical adenocarcinoma. [4]. Treatment modalities range from cone biopsies to simple and radical hysterectomy with or without pelvic lymph node dissection and pre-postoperative radiation therapy [5–7]. Conflicting conclusions on the prognosis of villoglandular adenocarcinoma have been reported in various case series, most of them quoting excellent prognosis [1, 8, 10] with few reporting rapid deterioration [9–11]. The standard treatment for advanced adeno-squamous cervical cancer is chemo radiation. As our patient had a stage IIB distal villoglandular papillary adenocarcinoma, fertility sparing surgery was not an option. External beam radiotherapy with concomitant cisplatin chemotherapy, was used which resulted in good response. The prognosis following surgical treatment is excellent. Surgical treatments described are cone biopsy alone (7 patients), simple hysterectomy (11 patients), and radical hysterectomy (57 patients). Disease-free periods have ranged from 13 months to 7 years. Only one death has been reported in the literature in a woman who had iliac node metastases in association with clinical stage 1B villoglandular papillary adenocarcinoma. Thirty-six months after a radical hysterectomy, she developed vaginal recurrence and died of the disease in 46 months. The usually good prognosis is also reflected by the early stage that most tumors associated with villoglandular papillary adenocarcinoma present, with over 94% of patients Having stage 1 disease [12]. The absence of lymphovascular invasion and nodal metastases is usual. Because of the inherent good prognosis of this tumor, fertility-conserving procedures have been suggested [1]. Young and Scully recommended that the initial treatment should be a cone biopsy if three criteria were fulfilled: (1) The margins of the cone are clear of the disease, (2) the depth of invasion is no more than 3 mm, and (3) there shall be no evidence of lymphovascular invasion on histology.

## Conclusion

Villoglandular papillary adenocarcinoma of the cervix is considered to have a favorable prognosis, and likewise, the treatment described is also conservative with close follow up; especially in young patients.
